# Progress in Medicine

**Published:** 1898-12-03

**Authors:** 


### Progress in Medicine.

### DIPHTHERIA.

The differential diagnosis of the diphtheria bacillus upon which so much depends in practice is not always an easy matter. Leedham Green,[1] in a communication on this subject, remarks that the whole difficulty lies in the separation of the true, or Löffler, bacillus from the various false or pseudo bacilli, and that our knowledge on the relationship of these micro-organisms is still imperfect. Bacilli which closely resemble the true diphtheria microbe are very widely distributed. Not only are they found in association with the true bacillus, but also in the normal secretion of the mouth and nasopharynx. These bacilli are also almost constantly present on the conjunctiva, both in health and disease. The bacillus of xerosis conjunctivæ is one of the micro-organisms which, like the Von Hofmann-Wellenhof bacillus, so closely resemble the diphtheria microbe as to render the diagnosis of the latter a great difficulty. It is quite impossible merely from the appearance of these organisms to distinguish one from the other. Although generally the diphtheria bacillus is long, slender, and frequently clubbed at one or both ends, and the pseudo-bacillus is short, straight, and regular, this is by no means always the case. The characteristic of the diphtheria bacillus is its great variability, now long, now short, now straight, now curved. The production of acid by the true bacillus when grown in broth Green shows is unreliable as a distinguishing test. The hope that specific agglutination of an antitoxin serum would prove a reliable distinction has been shown to be fallacious. Even inoculation of guinea-pigs is not always absolutely reliable for the purpose.

But the one test which Leedham-Green at present believes is as accurate as it is simple, is the staining of the polar granules by the Neisser method. Bates and Ernst drew attention to these spore-like bodies, and Max Neisser, who first recognised their diagnostic value, found that if the diphtheria bacillus were grown on solidified Löffler's blood serum, the bacilli when treated first with a dilute acid solution of methylene blue, and subsequently with Bismark brown, appear as slender rods stained brown, containing dark, violet-coloured polar granules at either end. In order that this method should be trustworthy, it is necessary to pay attention to several details. According to Neisser, the culture must always be grown on solidified blood-serum at a temperature of 35 deg. C.; certainly not higher than 36 deg. C., and the growth should be examined when from ten to twenty hours old. If examined earlier the staining may be limited to only a few bacilli; on the other hand, if older than twenty hours there is a danger of other bacilli besides the diphtheria showing somewhat similar staining. During the twelve months preceding this paper of Green's this method had been adopted at the bacteriological laboratory of Mason's College, and there was abundant opportunity for testing the efficiency of the stain, and Green has found it of the greatest value. He says it is simple, rapid, and trustworthy. But the stain must be freshly prepared, or the blue quickly loses its power and the polar granules are not stained. He makes up both the stains fresh every week. Some little practice is required to get the maximum staining. He has occasionally found that where the first film showed few or no polar granules, a second trial showed them clearly. The polar granules, which are more oval than round, are, as a rule, found at one or both ends of the bacillus. Occasionally there is a third situated towards the middle, and more rarely a fourth is seen. As the bacilli are very faintly stained, a good microscope, plenty of light, and accurate focussing are essential.

Green does not think this double stain is accurate enough to decide once and for all between the true and the false varieties of bacilli. Green believes that the polar granules are the result of a degenerative process, and he regards the positive side of Neisser's reaction as of greater value than the negative. If the suspicious micro-organism stains with characteristic polar granules then it is certainly diphtheria; if, on the other hand, no polar granules are present, then most probably it is not diphtheria. Finally, Green remarks that for purposes of diagnosis the Neisser's stain should be supplemented by an examination of "the hanging drop" and staining with Gram.

Two cases of noma in which diphtheria bacilli were found, and in each of which recovery took place on treatment by diphtheria antitoxin, are recorded by Freymuth and Petrowsky.[2] One was in a boy aged eight. The alveolar process of the right half of the lower jaw was involved after the right cheek, right half of the upper and lower lips, and sub-maxillary regions were attacked. The boy was unconscious and supposed to be dying when the serum was injected, 9,500 units being given in twelve days. The micro-organisms which could be cultivated were staphylococcus aureus, bacillus Löffler, and the pseudo-bacillus diphtheriæ. In the smear preparation were found numerous spirillæ and plump, slightly curved bacteria, which were diagnosed as those commonly found in carious teeth.

**Value of the Culture Test.**—Delépine[3] reports some interesting results bearing on the agreement observed in his laboratory between the laboratory results and the further clinical study of the cases. Thus, of 311 specimens taken from Manchester cases, in 89 diphtheria bacilli were found, while in 218 no diphtheria bacilli were found. But their negative report was not accepted by the medical attendant in 20 of these 218 cases. Excluding four doubtful cases, there were 307 cases; the bacteriological report was openly accepted in 188 cases, received without opposition in 99 cases, and challenged in 20 cases. Of these 20, three developed scarlet fever, one other probably had scarlet fever, one other was seen by Dr. Niven, who satisfied himself clinically that it was not diphtheria; another recovered so rapidly that it was difficult to support the diagnosis of diphtheria on any clinical grounds. Of the remaining 14 cases to be accounted for, two developed diphtheria, but in none of the others was it clear that the cases were really diphtheria. But if it is admitted that in all the 14 cases the bacteriological diagnosis was inaccurate, it only gives 4 per cent. of error; while, on the other hand, out of 307 cases suspected of being possibly diphtheria, not less than 218 were bacteriologically not cases of that disease, or, excluding the 14 cases, 204 cases out of 293 suspected cases were affected with some other disease, *i.e.*, 69 per cent. of clinical error compared with 4 per cent. of laboratory error.

With Delépine's results we may compare those of University College Hospital, reported by Martin and Hunt.[4] Out of 178 cases, the diphtheria bacillus was found in pure growth or mixed with cocci in 149; in 18 cases the bacillus was not found on examination; and in 11 no bacteriological finding was recorded. A few of the cases in which the bacillus was not found were undoubtedly diphtheria. All were admitted on clinical grounds as diphtheria.

1 Birmingham Med. Rev., Oct., 1898. 2 Deut. Med. Woch., No. 38, Sept., 1898; Treatment. Nov. 10, 1898. 3 Lancet, Feb. 12, 1898. 4 Brit. Med. Jour., Sept. 3, 1898.

